# Effectiveness and implementation of an intervention strategy-based health promoting schools framework to reduce idiopathic scoliosis and myopia among children and adolescents in Shanghai, China: study protocol

**DOI:** 10.1186/s12889-026-28184-5

**Published:** 2026-07-09

**Authors:** Tongtong Zhang, Xin Li, Juping Liang, Qimeng Fan, Xiaoyan Yang, Huan Lu, Haiyan Yuan, Fan Wu, Yongfang Huang, Xueli Du, Qing Du

**Affiliations:** 1 Chongming Hospital, Shanghai University of Medicine and Health Sciences, Shanghai, 202150‌ China; 2 Department of Rehabilitation, Xinhua Hospital affiliated to Shanghai Jiao Tong University School of Medicine, Shanghai, 200092 China; 3 Children’s Rehabilitation Innovation and Transformation Research Center of Yuanshen Rehabilitation Institute, Shanghai Jiao Tong University School of Medicine, Shanghai, 200025 China; 4 Shanghai Chongming District Center for Disease Control and Prevention, Shanghai, 202162 China; 5 Shanghai University of Medicine and Health Sciences, Shanghai, 201318 China

**Keywords:** Children and adolescents, Idiopathic scoliosis, Myopia, Health Promoting Schools, Cluster randomized controlled trial

## Abstract

**Background:**

Idiopathic scoliosis (IS) and myopia have emerged as major public health concerns affecting children and adolescents worldwide, with a confirmed comorbid association. Both conditions share common risk factors, including outdoor activity, sedentary behaviour, screen time, and sleep duration. This protocol describes a study designed to evaluate the effectiveness of the “ATPISM” intervention strategy, which is developed based on the six core characteristics of the Health Promoting School (HPS) framework advocated by the World Health Organization (WHO). Appropriate Techniques for the Prevention of Idiopathic Scoliosis and Myopia (ATPISM) aim to establish a comprehensive preventive intervention strategy against idiopathic scoliosis and myopia within the HPS framework, and to assess and explore the implementation of ATPISM across five dimensions: acceptability, feasibility, adoption, usage, and maintenance.

**Methods and analysis:**

This is a two-phase hybrid type I effectiveness implementation trial. A 1:1 parallel-group cluster randomized controlled trial will be conducted in 30 schools (15 intervention, 15 control) across three districts in Shanghai, involving approximately 5964 students. The primary outcomes will include differences in IS and myopia screening positivity rates, body composition and behavioral indicators among children and adolescents. Secondary outcomes will assess the Reach, Effectiveness, Adoption, Implementation, and Maintenance of the ATPISM intervention using the RE-AIM framework. To account for school clustering effects, mixed-effects models will be developed to compare group differences. Subgroup analyses will be conducted to identify interaction effects (stratification factor×group) and examine differences in intervention effects across strata. Additionally, Difference in Differences (DID) analysis will be employed.

**Discussion:**

This study is the first to apply the HPS framework for concurrent prevention of IS and myopia in China. The ATPISM strategy, centered on schools and supported by families and communities, is expected to reduce IS and myopia prevalence and improve health behaviors. Findings will provide evidence-based insights for scaling up HPS in China and offer a replicable model for global adolescent health promotion, safeguarding the physical and mental well-being of children and adolescents.

**Trial registration:**

ChiCTR2600117433. Registered January 23, 2026.

**Supplementary Information:**

The online version contains supplementary material available at https://doi.org/10.1186/s12889-026-28184-5.

## Introduction

Scoliosis and myopia have become increasingly serious health issues among children and adolescents. Scoliosis is characterized by a three-dimensional spinal deformity involving lateral curvature of one or more spinal segments, accompanied by vertebral rotation and changes in the sagittal plane. Idiopathic scoliosis (IS) is one of the most common types, with a prevalence of 1.7% (95% CI: 1.1%-2.4%) among children and adolescents [1, 2]. Mild cases are often overlooked due to an absence of symptoms, while progression to severe IS (Cobb angle ≥ 40°) can result in cosmetic deformities, back pain and other symptoms. This can have serious social and psychological consequences, including depression and anxiety. Furthermore, IS can diminish the quality of life for patients and their families while increasing economic burdens [3]. The prevalence of myopia and high myopia continues to rise globally and is expected to affect nearly 5 billion and 1 billion people, respectively, by 2050 [4]. Myopia onset is becoming more prevalent in younger generations, and younger age at onset increases the risk of progressing to high myopia [5, 6]. High myopia is often accompanied by pathological ocular changes or complications that can lead to visual impairment or even blindness [7]. Previous findings suggest that there may be an association between IS and myopia [8]. A systematic review and meta-analysis documented visual problems associated with scoliosis, providing moderate evidence of an association between ocular conditions, such as severe visual impairment, strabismus, myopia, and anisometropia, and scoliosis. Patients diagnosed with ocular conditions were approximately three times more likely to develop IS than individuals in the control group without ocular conditions [9]. This finding is consistent with the conclusions of a study conducted in Nantong, China [10].

It is widely acknowledged that children and adolescents are in the peak of their growth and development. This period is characterized by significant changes that are both physical and psychological in nature. The external environment plays a crucial role in their physical development. Despite the ambiguity surrounding the etiology and pathogenesis of IS, it is an established fact that both IS and myopia are influenced by genetic and environmental factors [5, 11].

These conditions have been shown to share common risk factors, including outdoor activity, sedentary behavior, screen time, and sleep duration [6, 12, 13]. In the majority of cases, the most prevalent childhood and adolescent diseases, including IS and myopia, rarely manifest in isolation. Interventions designed to prevent multiple diseases, including IS and myopia, may be more efficacious due to the presence of shared or analogous public health risk factors. Moreover, previous research has demonstrated substantial overlap between prevention and intervention strategies for IS and myopia (e.g., outdoor activities and screen time [14, 15], school-related factors [16, 17], and sleep duration [18, 19]. In China, IS and myopia have been designated as priority health issues in the monitoring of common diseases among primary and secondary school students [20], with structured prevention guidelines and intervention protocols respectively [21, 22]. Nevertheless, a comprehensive and integrated intervention strategy for preventing both conditions is still lacking.

The dissemination of educational information pertaining to spinal and visual health can be sustained throughout the school-age period. The period of childhood and adolescence is of particular significance in the context of public health, as it is a time when health promotion exerts a substantial influence on future health outcomes. The adoption of healthy behaviors and attitudes during this phase of life often continues into adulthood, thereby shaping the trajectory of health over the long term [23]. In consideration of this pivotal stage, educational institutions, as primary settings for the daily lives of children and adolescents, function as vital venues for health promotion, thereby possessing considerable potential to deliver intensive, long-term, and large-scale health programs to this population [24]. The Health Promoting Schools (HPS) initiative constitutes the optimal platform for the instilling of healthy habits during the formative early years. The HPS framework, developed in 1996 based on the guiding principles of the Ottawa Charter for Health Promotion and recommendations from the WHO Expert Committee on Comprehensive School Health Education and Promotion, is grounded in the interrelationship between health and education, supported by Bronfenbrenner’s ecological theory [25, 26]. The programme adopts an eco-holistic approach to the development of school environments that are conducive to the promotion of health and the facilitation of healthy behaviors [27].

The HPS framework currently lacks a precise definition. Existing research considers the HPS framework from three perspectives: (1) formal health education curricula, (2) the school ethos and environment, and (3) engagement with families and communities [28]. The HPS framework emphasizes the establishment of foundational health education curricula, as well as recognizing the importance of school-based health strategies, linkages to health services, and collaborative relationships among schools, families, and communities. The HPS framework has been adopted and implemented in many countries worldwide in a number of areas, including infectious diseases [29], obesity [30], oral health issues [31], nutrition [32], diet and physical activity [33]. A Cochrane systematic review found evidence of positive health impacts from HPS, suggesting that the framework can promote certain health outcomes by evaluating its effectiveness in relation to children’s health and well-being [34].

This protocol proposes a comprehensive intervention strategy “Appropriate Techniques for the Prevention of Idiopathic Scoliosis and Myopia (ATPISM)”, to reduce the high prevalence of IS and myopia among children and adolescents. It is based on the WHO advocated HPS framework, combined with the implementation guide for making every school a health promoting school [35], the WHO guidelines on school health services [36], and the global standards and indicators for making every school a health promoting school [37], which were jointly released by the WHO and the United Nations Educational, Scientific and Cultural Organization (UNESCO) in June 2021. To gain deeper insights into optimizing intervention measures and implementation processes to facilitate dissemination, the protocol will be embedded within the RE-AIM (Reach, Effectiveness, Adoption, Implementation, Maintenance) framework for process evaluation [38]. The research objectives are: (1) To evaluate the differential effects of the ATPISM intervention on positive screening rates for IS and myopia, body composition parameters, reading/writing posture, outdoor activity, screen time, backpack habits, sleep duration, out-of-school visual habits, self-rated depression and anxiety scores among children and adolescents; (2) To evaluate the Reach, Effectiveness, Adoption, Implementation, and Maintenance of the ATPISM intervention using the RE-AIM framework.

## Design and methods

**Fig. 1:**
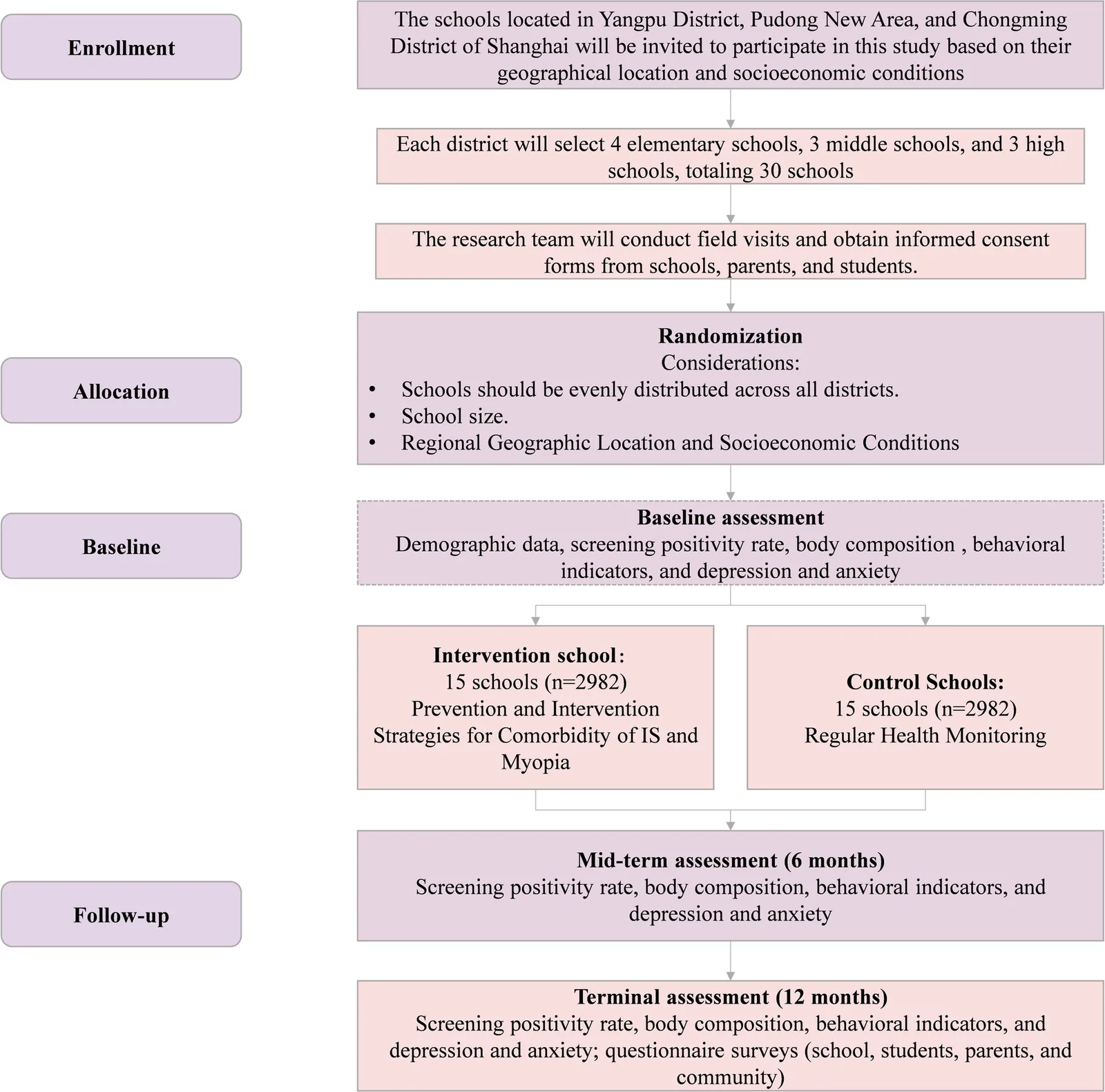
Trial implementation process

The study is a two-phase hybrid type I effectiveness implementation trial, aiming to determine its efficacy and explore contexts for routine implementation. In the first stage, a comprehensive intervention strategy- ATPISM -based on the WHO-advocated HPS framework will be designed to evaluate its effectiveness in reducing the incidence of IS and myopia in children and adolescents. In the second phase, the RE-AIM framework will be employed to evaluate the implementation with regard to acceptability, feasibility, adoption, usage, and maintenance of the ATPISM intervention strategy. Figure 1 provides an overview of the intervention strategy. Additionally, this study adhered to the SPIRIT guidelines for clinical trial protocols [39] (Supplementary Material).

### Effectiveness evaluation

#### Study setting

**Fig. 2:**
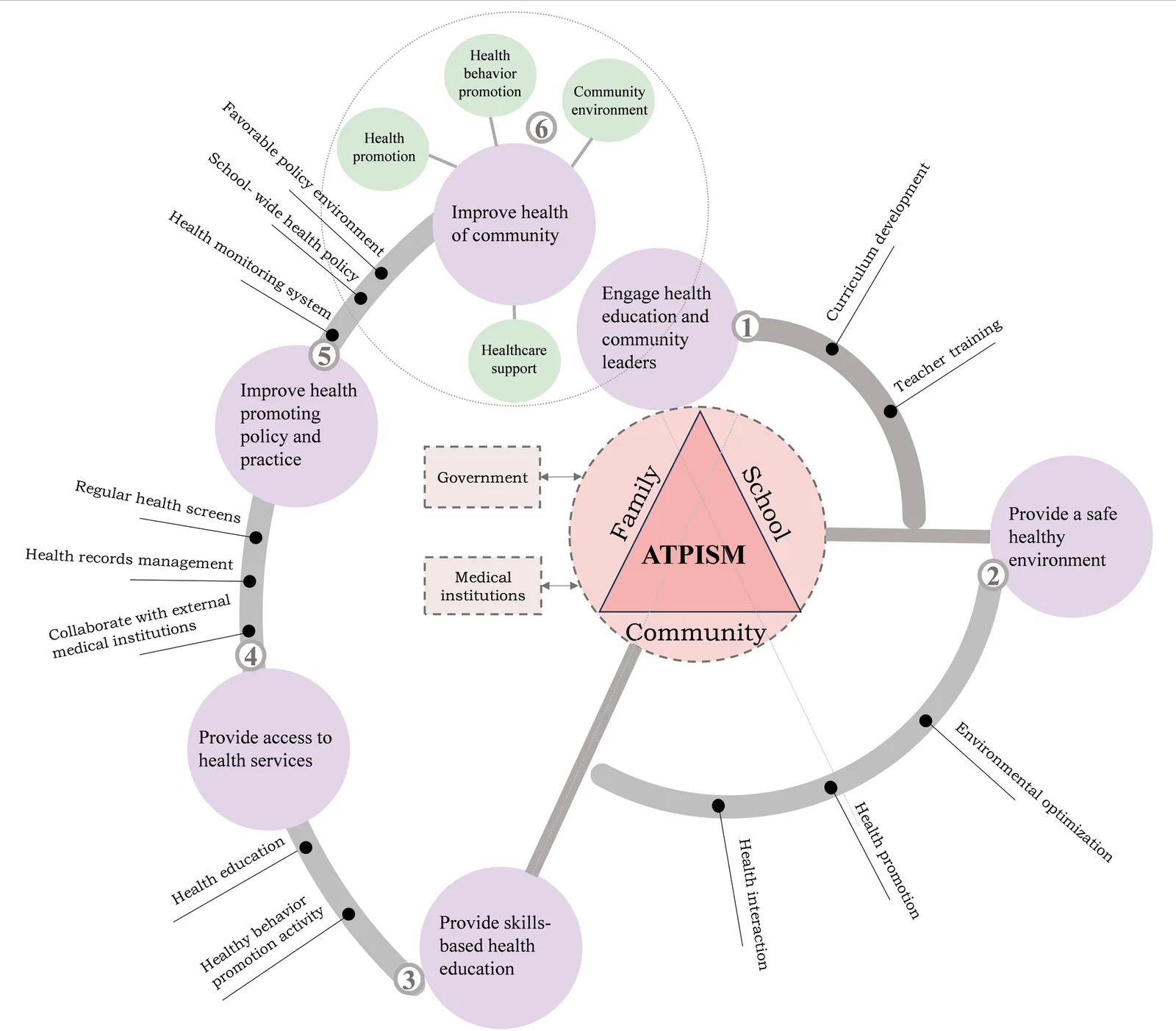
The flow chart of participants in the trial

A parallel group design will be implemented in Yangpu District, Pudong New Area, and Chongming District based on Shanghai geographical location and socioeconomic conditions, and a 1:1 allocation double-group cluster randomized controlled trial will be conducted. A total of 30 schools will be selected, with four primary schools, three middle schools and three high schools from each district. Fifteen of these schools will be assigned to the control group and the remaining fifteen to the intervention group. The intervention programme will be implemented over 12 months for enrolled students in grades 1 to 4 of primary school, grade 1 of middle school, and grade 1 of high school during the fall semester of 2026 (aged 6–18) to prevent loss to follow-up due to school transitions (see Fig. 2). All intervention schools across the three districts will follow a standardized, unified research protocol.

### Randomization and blinding

The study will be randomized at the school cluster level, with 30 schools being randomly assigned at a ratio of 1:1 to receive either the ATPISM intervention or the conventional intervention. To minimize the potential for selection bias, the clusters will be identified and recruited prior to randomization. The allocation sequence will be generated using a computer-generated random sampling method. Schools will be invited to participate in the project in the order determined by this list. If the selected schools decline to participate, the next school of the same size will be recruited from the random list. To achieve allocation concealment, randomization will be performed by an independent researcher who will not be involved in the recruitment process. Due to the nature of the intervention, it was not feasible to blind participants and intervention staff. Assessors will be blinded in this study.

### Sample size

Previous studies have reported an average co-prevalence rate of 2.592% for IS and myopia among children and adolescents in China, which was calculated as the weighted mean of co-prevalence data from five representative regions (Beijing, Guangzhou, Inner Mongolia, Shantou, Tianjin) in Table 1. The weighting was adjusted by the sample size of each regional study to reflect the demographic distribution of primary and secondary school students in Shanghai. This value was selected to balance the regional variability of co-prevalence and ensure the statistical power of the trial in the Shanghai context. Using the following formula, it was calculated that this study should include 5,964 children and adolescents in the intervention group, with statistical power of 90%, a significance level of 5%, an expected reduction in prevalence of 50%, and a dropout rate of 20%.

$$\:\mathrm{n}=\frac{2\stackrel{-}{\mathrm{p}}\stackrel{-}{\mathrm{q}}{({\mathrm{Z}}_{\alpha\:}+{\mathrm{Z}}_{\beta\:})}^{2}}{{(\mathrm{p}1-\mathrm{p}2)}^{2}}$$

**Table 1:** Co-prevalence of idiopathic scoliosis and myopia in children and adolescents

Characteristic of Population	Years (sample size)	Co-prevalence of IS and Myopia (%)	Country/Region
Primary and secondary school students	2019(34923), 2020(34321), 2021(36008), 2022(35598)	0.69-2.31	Beijing, China [40]
	2019(4248), 2020(4230), 2021(4266), 2022(25476), 2023(25587)	2.96-6.0	Guangzhou, China [41]
	2019(181533), 2020(141552), 2021(200987), 2022(190918)	1.14-1.95	Inner Mongolia, China [42]
	2019(1934), 2020(2131), 2021(2149), 2022(1969), 2023(2123)	3.9	Shantou, China [43]
	2023(41,645)	2.65	Tianjin, China [44]

### Eligibility criteria

#### School level

##### Eligibility criteria for schools

1. The school employs health-related staff, such as school nurses and teachers responsible for health education and physical education.
2. School administrators agree to participate in this study.
3. The total number of students per grade level must be ≥ 50. Most classes should have 30–50 students. If a class has fewer than 40 students, two classes will be recruited. If it has more than 40 students, one class will be recruited.

##### Exclusion criteria for schools

1. Boarding schools.
2. Ethnic minority and special education schools.
3. Those undergoing other similar programme focused on preventing IS and myopia.
4. Those with plans for merger, division or relocation during the implementation period of this study.

#### Classes level

The study will include primary school students in grades 1 to 4, as well as middle and high school students in their first years. Typically, these students will be between 6 and 18 years old. This age range has been chosen to take into account the greater academic pressure and subsequent educational advancement concerns faced by older students.

##### Eligibility criteria for classes

1. Students who will be in grades 1 to 4 of primary school, grade 1 of middle school, and grade 1 of high school during the fall semester of 2025.
2. Students must have the consent of their head teacher to participate.

#### Students and parents’ level

##### Eligibility criteria for students and parents

1. There are no plans to transfer schools during the study period.
2. Complete medical records are available for the student throughout their enrolment.
3. Parents have access to smartphones and participate in the class WeChat group (a Chinese social media platform).
4. Students and parents understand the study content and are willing to sign the informed consent form.

##### Exclusion criteria for students and parents

1. Presence of eye diseases, visual impairments (e.g., color blindness, color deficiency), or prior eye surgeries (e.g., laser procedures).
2. Scoliosis caused by identifiable conditions (e.g., congenital, postural, neuromuscular disorders, or other diseases such as neurofibromatosis, Marfan syndrome, osteogenesis imperfecta, metabolic or endocrine disorders).
3. History of psychiatric disorders or rheumatic diseases.
4. Presence of contraindications preventing participation in physical activities.
5. Received prior rehabilitation or surgical treatment for IS or myopia.

### Intervention

**Fig. 3:**
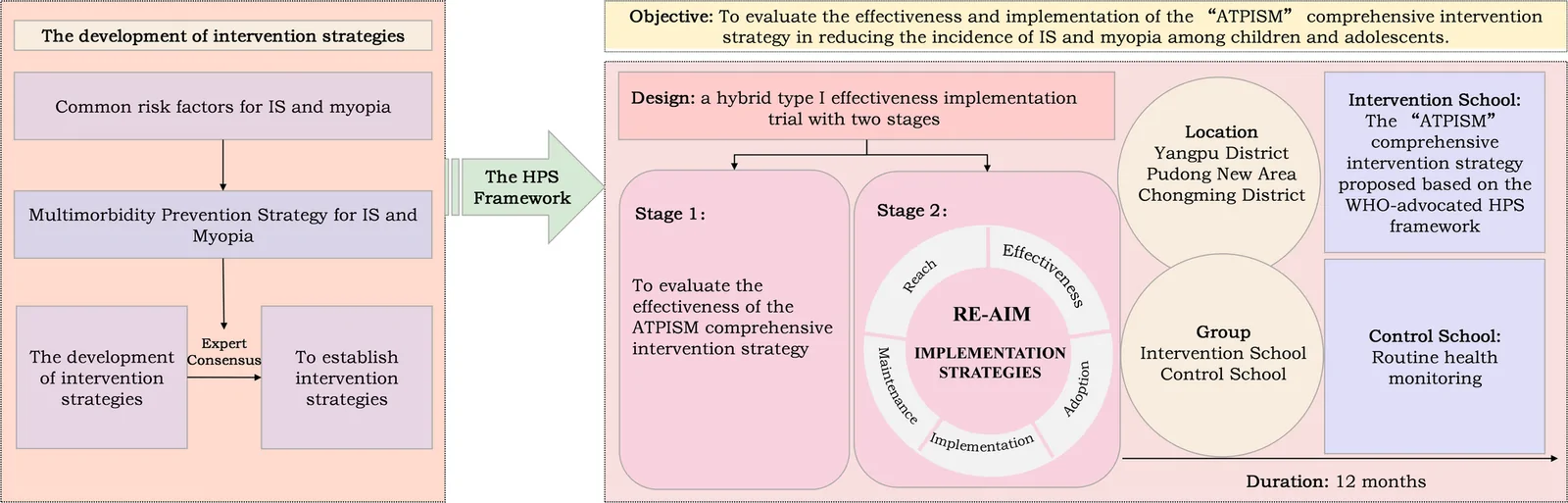
The “ATPISM” comprehensive intervention strategy developed based on the WHO promoted HPS framework

The WHO has proposed the HPS framework, which is aimed at supporting global schools in creating healthy and positive environments. The framework advocates for the establishment of schools that continually seek to strengthen their capacity to promote healthy living, learning and working. The HPS initiative: (1) promotes health education through formal school curricula, (2) modifies the physical and social environment of schools, and (3) recognizes and values the roles of families and communities in promoting children’s health. The current study proposes an ATPISM intervention based on the core features of the HPS framework to establish a IS and myopia prevention system among children and adolescents (see Fig. 3). Table 2 provides the timepoints of participation for the study subjects.

**Table 2:** Participant timeline according to SPIRIT

	Trial period			
	Enrollment	Allocation	Post-allocation	Close-out
Time point	December 2025 - January 2026	January - February 2026	February 2026 - February 2027	February - March 2027
Enrollment				
Eligibility screen	×			
Informed consent	×			
Randomization		×		
Interventions				
Intervention			×	
Control			×	
Assessments				
Anthropometric measure	×		×	×
Psychological measures	×		×	×
Questionnaire	×			×

### Intervention group

#### Engage health education and community leaders

The research team will assemble a curriculum development team comprising schoolteachers, health education specialists and professionals from relevant departments, including ophthalmology and rehabilitation medicine. This team will design health education courses tailored to students at different levels of education, with a focus on preventing IS and myopia. Each 40-minute session will be conducted every 2–3 weeks, with 6 sessions per semester totaling 12 sessions. Once the health education curriculum has been designed, training will be provided for teachers, school staff and community workers participating in the project. This training will incorporate theory, practical sessions, and workshops to ensure the effective implementation of health education activities. Conduct a pre-test on spinal and visual health knowledge for all participating teachers, only those with a score ≥ 80% can participate in formal training. Teachers must pass a practical exam and a teaching demonstration to obtain a health guidance certification by the research team, and conduct quarterly unannounced classroom observations and knowledge spot-checks, organize monthly inter-school sharing sessions for teachers to address implementation difficulties. Teachers with substandard performance will receive one-on-one retraining within 2 weeks.

#### Provide a safe healthy environment

This dimension encompasses various aspects, such as the design of school physical environment, campus activities, and daily interactions between teachers and students. It aims to comprehensively create an environment that helps students maintain proper posture and reduces adverse effects on the spine and eyes, thereby playing positive roles in preventing IS and myopia.

#### Provide skills-based health education

Schools provide targeted health education courses for children and adolescents on how to prevent IS and myopia. These courses aim to equip students with the necessary knowledge and skills. Schools collaborate with professionals will be launch long-term advocacy activities focused on preventing IS and myopia, helping students to develop good health habits. Health education lectures for parents will be held once per semester, providing them with methods for supervision and guidance within the home environment. Teachers and parents conduct monthly 15-minute online meetings to exchange students’ performance at school and home. The research team compiles a family supervision effectiveness ranking and shares excellent cases to motivate participation. The research team will review records monthly and provide personalized feedback.

#### Provide access to health services

Health screenings will be conducted once per semester to establish a regular process for identifying IS and myopia. Health records will be created for each student. These records will be reviewed and analyzed annually in collaboration with external medical institutions. Based on the findings, targeted guidance or medical advice will be provided. Separate health monitoring systems are in place for healthy individuals, individuals with suspected conditions, and individuals with confirmed diagnoses.

#### Improve health promoting policy and practice

1. Policy level: The objective is to provide schools and society with replicable guidelines, standards, and policy frameworks for preventing spinal problems and visual impairments among children and adolescents. This will further enhance societal awareness of these health issues. Additionally, education authorities should oversee schools to ensure the effective implementation of prevention measures.
2. School Level: Schools will integrate IS and myopia prevention into their comprehensive health policies, teaching curricula, and activities, and ensure the consistent execution of these initiatives.

#### Improve health of community

The school will serve as a connecting point to establish links with parents and the broader community, building a comprehensive “school-home-community” health network. Embed IS and myopia prevention into existing community programs such as “Shanghai Family Health Promotion Month” and “Youth Summer Care Camps”. Two community health education events will be organized each semester, where community professionals and school teachers will educate parents about preventing IS and myopia. It will also increase outdoor activity time by sharing community facilities and planning designated exercise areas within the community. Each participating school will assign a dedicated community liaison teacher to coordinate with the community’s cultural and sports department. A joint scheduling system will be established to reserve community facilities for students from 16:00 to 18:00 every Monday, Wednesday, and Friday. The community will designate 2–3 staff trained by the research team to supervise equipment use, while school teachers will lead spinal health exercise courses twice a week. A registration system via the class WeChat group will be implemented to track student participation, with a minimum attendance requirement of 80% for intervention compliance.

### Control group

Only periodic health monitoring will be provided for children and adolescents in the 15 control group schools. For ethical reasons, these schools will receive the same educational materials as the intervention schools after this study has been completed.

### Quality control

This study involves the following groups: medical institutions (ophthalmology and rehabilitation personnel), schools (principals, health and physical education teachers, school physicians, etc.), students, parents, community leaders and staff, and education authorities.

The study will begin with introductory meetings for project team members, including school and community leaders, as well as relevant personnel from the education department. These sessions will cover the epidemiology of IS and myopia, the study design and the methods of data collection. Team members will receive a study manual clarifying the responsibilities and division of labor for different implementers. Prevention measures for IS and myopia among children and adolescents will be implemented at four levels: schools, students, parents and the community. Table 3 provide detailed overviews of the intervention content for each implementer group.

**Table 3:** The detailed overviews of prevention and intervention for the comorbidity of idiopathic scoliosis and myopia

Content	Frequency and duration	Concrete measure	Executives
1. Engage health education and community leaders			
Collaboration on curriculum development	Health education sessions on the prevention and control of IS and myopia will be conducted every 2-3 weeks, each lasting 40 minutes, with 6 sessions per semester and a total of 12 sessions.	The curriculum development team will be comprised of school teachers, school nurses, health education experts, and professionals from ophthalmology, rehabilitation, and other related disciplines.(1) The basic course content: the physiological structure of the spine and eyes; the causes, symptoms and hazards of IS and myopia; and the relationship between healthy lifestyles and spinal and eye health.(2) The teaching aids will be used in a variety of methods and technologies to assist with teaching and learning. For example, VR will be used to create vivid health education courseware. An interactive online course platform will be developed where students can conduct virtual spine and eye health check-ups, observe 3D animations demonstrating correct eye and sitting habits and participate in health quiz games.(3) Primary school students (grades 1-4) will be taught health knowledge through stories, children’s songs, and other simple, visual forms. Middle school students in the first year will receive in-depth explanations of the scientific principles of disease prevention and treatment, and their awareness of independent health management will be cultivated.	Medical institutions and selected schools
Teacher training	(1) The training will include lectures on theoretical knowledge, practical demonstrations, and communication and discussion sessions. There will be 2-3 centralized training sessions per semester, each lasting 8 hours, totaling 4-6 centralized training sessions.(2) One online learning task will be assigned per month, with each task taking one hour to learn the prevention and control guidelines, totaling 12 hours of learning.(3) Inter-school health teaching seminars: one inter-school health teaching seminar will be held per semester, one hour each time, with a total of two seminars.	Professionals from relevant disciplines will be invited to provide training for teachers and community workers:(1) Theoretical knowledge lectures will include how to identify the early symptoms of IS and myopia, and how to provide personalized health guidance according to the characteristics of different students.(2) Practical exercises: Simulated scenarios will be conducted, and participants will be taught to use vision charts and Scoliometers for daily health monitoring.(3) Online learning: A health knowledge update mechanism will be established, where teachers and community workers will be regularly provided with the latest guidelines on scoliosis and myopia prevention and control by professionals.(4) Inter-school health teaching seminars will provide opportunities for teachers to share health teaching experiences with colleagues from other schools and exchange successful methods of teaching health knowledge in the classroom and strategies for encouraging healthy behaviors.	Program members, school teachers, and community workers
2.Provide a safe healthy environment			
Optimization of the school physical environment	At the beginning of each semester, a comprehensive inspection of the height of desks and chairs, as well as lighting and illumination conditions, will be conducted. Regular checks on the safety of sports venues and equipment will be carried out, and full greenery coverage on campus will be ensured.	(1) Schools will regularly adjust the height of desks and chairs and will promote the use of height-adjustable furniture. The height of desks and chairs will be adjusted to match students’ heights, ensuring that when students sit on the chairs, their feet will be able to rest flat on the ground, their thighs will be parallel to the floor, and their eyes will be at a suitable distance from the desktop (about 30-35 cm).(2) Lighting requirements: The illuminance on desktops will be no less than 300 lx, and that on blackboards will be no less than 500 lx. Anti-glare devices will be installed on desktops to shield against glare from external light sources, which students will be able to activate when necessary.(3) Teaching and homework assignment will not rely on electronic products, and the use of electronic products for teaching will not exceed 30% of the total teaching time.(4) Small lockers will be provided to prevent schoolbags from occupying desk and chair space and interfering with students’ sitting posture. These lockers will be used to store textbooks and other items that do not need to be taken home, thereby reducing the weight of students’ schoolbags.(5) Safe and diverse physical activity venues will be established, including adequately sized playgrounds and sufficient sports training equipment.(6) Appropriate green plants will be added to the school campus.	Program members and selected schools
Campus health promotion activities	The healthy start week series will consist of two events that will be held at the beginning of each semester.	A series of activities will be organized on the themes of IS and myopia prevention and control:(1) During the health promotion campaign, an exhibition on health knowledge will be organized. Professionals from relevant disciplines will guide the content of the exhibition, which will cover topics such as the developmental processes of the spine and eyes, global trends in the incidence of IS and myopia, and the latest prevention and control techniques. Various forms will be adopted for the exhibition, including illustrations, physical displays (e.g. models of the spine and eyes), and multimedia presentations (e.g. relevant popularization videos). Volunteers will be arranged to provide explanations and guidance during the exhibition.(2) Health competitions covering all aspects of IS and myopia will be organized, with teams formed on a class basis and professionals will act as judges to review the answers provided.(3) Health speech contest: Students will be encouraged to prepare speeches on the prevention and control of IS and myopia.(4) A health promotion corner will be set up, and health tips and healthy living case studies provided by professionals will be updated weekly. Campus radio and other media platforms will be used to broadcast health promotion audio or video programmes during class or lunch breaks, once a day for 5–10 minutes each time.	Program members and selected schools
Mechanisms for healthy teacher-student interaction	Throughout the intervention.	(1) Teachers will actively encourage students to develop healthy habits as part of their daily teaching and management. For example, students will be reminded in the classroom that they will adjust the light intensity, maintain a suitable distance and normal sitting and standing postures when reading, doing homework and using electronic products; and that they will read and write with one fist, one foot and one inch between them and their work (eyes will be one foot away from books [about 30 cm], body one fist away from desk [about 6 cm] and fingers one inch away from pen tip [about 3 cm]).(2) Teachers will remind students to perform eye relaxation activities at regular intervals. They will establish requirements for recess activities and will ensure that students will perform relaxation exercises for their shoulders, neck, waist, back and limb joints during breaks. Teachers will arrange for a designated person to supervise these activities and will ensure that students will go outside during breaks to bathe in sunlight.(3) Teachers will establish a mechanism for interaction with students and will encourage them to provide feedback on any vision or spinal health issues they may have. Teachers will provide timely guidance and advice. The length of each interaction will depend on the specific situation, but it will ensure that students’ problems can be solved effectively.	Teacher
3. Provide skills-based health education			
Health education program	Health education sessions on the prevention and control of IS and myopia will be conducted every two to three weeks. There will be six sessions per semester and a total of 12 sessions. Each session lasts 40 minutes.	IS prevention:(1) Body posture: One lesson will be arranged to teach proper posture (standing, sitting and walking).(2) Posture self-checking skills: Two lessons will be arranged to teach students how to check if the spine is normal. For example, students will learn to perform the simple Adam’s test to check if the two sides of the spine are symmetrical.(3) Spine care exercises: Three lessons will be arranged to teach students how to relax and exercise the joints of the neck, chest, waist, back and limbs. This exercise routine will be promoted during recess activities, with the aim of adding some symmetrical back muscle group training to daily exercise, such as planks.Myopia prevention:(1) Eye hygiene knowledge: Three hours will be devoted to explaining the importance of eye hygiene and specific measures in detail.(2) Eye relaxation techniques: One lesson will be arranged to teach students relaxation techniques for their eyes, such as the 20-20-20 rule. This rule will involve looking at a near object for 20 minutes, then looking 20 feet (about 6 meters) away for 20 seconds.(3) Proper use of electronic products: Two lessons will be spent teaching students how to use electronic products properly, including how to adjust screen brightness and contrast, how to maintain an appropriate viewing distance and how to control the time spent using them (no more than 30-45 minutes at a time).	Medical institutions and selected schools
Healthy behavior advocacy activities	Throughout the intervention.	Together with professionals, we will launch a series of long-term health behavior advocacy activities for IS and myopia prevention and control.(1) Correct sitting posture challenge: Professionals will formulate detailed sitting posture assessment standards, including the correct position of the head, neck, shoulders, back, waist and legs. Schools will organize teachers to regularly check and record students’ sitting posture in class. Students who will maintain good sitting posture during the activity period will be praised and rewarded. Sitting posture checks will be carried out two to three times a week.(2) One hour of outdoor activities every day: Professionals will provide scientific guidance on outdoor activities. Schools will reasonably arrange outdoor activity time, integrating physical education classes, recess and extracurricular activities, to ensure that students will have enough time for outdoor activities every day (at least one hour). Parents will be encouraged to actively participate in their children’s outdoor activities at weekends and during the holidays, to promote home-school collaboration.(3) The 20-20-20 Rule will be practiced monthly to promote healthy eye use. Teachers will remind students to follow the rule in class and students will keep records of their progress.	Medical institutions and selected schools
Parent health education	One parent health education seminar will be held per semester, lasting 60-90 minutes, totaling two sessions.	With a focus on preventing IS and myopia, as well as providing guidance on home supervision, parents and students will be invited to attend together.(1) Parents will supervise their children’s eye habits and posture at home and ensure that they do not lie down while reading or using electronic devices.(2) Parents will be trained to recognize changes in their child’s vision, take standardized photos of their child’s torso, and observe and compare changes in their child’s physical appearance on a regular basis.(3) Good indoor lighting will be ensured; the height of desks and chairs will be regularly adjusted to fit students’ growth and developmental changes; suitable school bags will be chosen; and backpacks that distribute weight as evenly as possible on both sides will be recommended.(4) Parents will provide weekly feedback to teachers on their children’s performance at home. Teachers will then give parents further guidance and suggestions based on this feedback.(5) Nutritionally balanced meals that promote healthy development and are beneficial to students’ bone and eye health will be provided to students.	Medical organizations, selected schools and parents
4. Provide access o health services			
Regular health screenings	Health screenings will be held twice per semester.	Screening for abnormal spinal curvature and vision monitoring will be incorporated into the physical examination programme. For students found to have abnormalities, their information will be recorded and a re-examination will be arranged within one week. Appropriate advice will be provided based on the results of the re-examination.	Medical institutions and selected schools
Health records management cooperation with external medical institutions	Relevant information will be entered into the file promptly after each screening, review or intervention. Health records will be organized and analyzed once per school year to identify trends in IS and myopia incidence across the school population.	A health file will be established for each student, which will include basic information, the results of IS and myopia screening, re-examination records, personalized health guidance programmes, implementation status, etc., ensuring that each student has one file and that changes in health status are reflected in real time.	
5. Improve health promoting policy and practice			
Favorable policy environment	Throughout the intervention.	(1) Guidelines, standards, and policies for the prevention and control of spinal and vision health issues in children and adolescents will be promoted for use.(2) Relevant entities will actively guide medical institutions, schools, families, and the media to pay attention to spinal and vision health as well as prevention and control measures.(3) A joint prevention and control network involving families, schools, the wider community, medical and healthcare institutions, and relevant government departments will be established to ensure the implementation of three-tier prevention and control measures for spinal and vision health among children and adolescents.	Government department
School health policy	Throughout the intervention.	(1) Schools will incorporate IS and myopia prevention into health policy system and will define the school’s objectives in promoting students’ spinal and vision health.(2) Schools will publish an updated wellness policy at the beginning of each semester and will publicize it to all students, teachers, and parents.(3) Educational programmes or activities related to spinal and vision health, such as physical exercise sessions and health talks, will be organized. These courses and activities will be included in the regular teaching programme and will not be changed arbitrarily. A system will be set up to prevent the diversion of classes and avoid the occupation of physical education classes.	Medical institutions and selected schools
Improving the health monitoring system	Throughout the intervention.	(1) For people with normal spines, an environment conducive to spinal and visual health will be created. Spinal care exercises or movements targeting spinal core muscle groups will be actively carried out. Exercise, relaxation and stretching movements for the cervical, thoracic, lumbar and dorsal muscles will be increased. These practices will be designed in accordance with the current learning and living characteristics of children and adolescents. For people with normal eyesight, schools, families and communities will collaborate to increase daytime outdoor activities through various means. The aim will be to reduce the incidence of myopia and poor visual acuity and improve the visual environment.(2) Children with suspected spinal curvature anomalies will be guided to professional medical institutions for diagnosis and treatment. Appropriate treatment plans will be selected based on the severity of the condition, risk of progression and pathophysiological characteristics. These plans may include physical training, corrective gymnastics for spinal curvature anomalies, brace-wearing or surgical treatment. Treatment plans will be adjusted through regular follow-ups as prescribed by doctors. For those with insufficient hyperopic reserve or decreased visual acuity, targeted interventions will be implemented to improve their visual function. Since insufficient hyperopic reserve or reduced naked-eye vision may indicate abnormal visual function, these individuals will be advised to undergo refractive examinations (e.g. medical optometry) in medical institutions for a clear diagnosis and timely corrective measures.(3) For those with abnormal posture, they will be advised to correct their posture and eliminate high-risk behaviors. School doctors, teachers or parents will conduct long-term monitoring based on spine protection exercises. This will involve taking monthly back photos to check for abnormal signs, and those affected will be arranged for timely re-examination. If necessary, some assistive devices will be used to maintain normal posture under the guidance of healthcare professionals. Those who have already developed myopia will be referred to medical institutions to select appropriate glasses as prescribed by doctors. Primary and secondary school students will undergo vision checks at medical institutions every 6 to 12 months. If their visual acuity with glasses deteriorates, they will be advised to consult a doctor about replacing their glasses.	Medical institutions and selected schools

During the implementation process, the project team, schools and community members must adhere strictly to the research manual. Two members of staff from the education department will assist with the implementation of the ATPISM intervention. Additionally, each school serving as a research site will have one designated coordinator to conduct the programme. The programme will utilize WeChat to create group chats for the project team, schools, community members and relevant education authorities. A real-time updatable document will be created, which the project team and the relevant Ministry of Education unit must review twice weekly, providing feedback to schools, parents and community members to ensure timely monitoring and follow-up of the implementation process. Furthermore, WeChat group chats will be set up to support the dissemination of IS and myopia prevention strategies, health education and daily supervision to parents and students.

Additionally, formal survey will be conducted after the intervention. This will use revised questionnaires for schools, students, parents and the community, based on the preliminary survey results and relevant previous research. To ensure the quality of the questionnaires, the survey staff will undergo uniform training and certification before deployment. Electronic questionnaires will be distributed via WeChat group chats and project coordinators at each school will be responsible for collecting and verifying the responses. Responses will be compiled into an Excel 2021 database, excluding those with excessive consistency or logical errors. Set up a WeChat feedback group for teachers, parents, and community workers to report implementation difficulties. The research team responds within 48 h and adjusts measures if necessary.

### Data collection

#### Qualitative data collection

Qualitative methods can be used to gather the views of professionals from related fields on the development of intervention measures for IS and myopia prevention. During the development of these strategies, the study will collaborate with experts from various domains, including education authorities, ophthalmology, rehabilitation medicine, school health services and school physical education, who specialize in the prevention of IS and myopia among children and adolescents. The aim of this collaboration is to gather insights from these specialists for formulating preliminary intervention strategies. The sample size for the qualitative study will be calculated based on the principle of dynamic saturation, meaning the sample size is considered saturated when no new information is obtained after the next interview [45]. Semi-structured interview guides will be developed based on the research objectives, covering four core dimensions: (1) Perceived feasibility of integrating IS and myopia prevention into school health programs; (2) Key barriers and facilitators for implementing multi-level interventions (school-family-community); (3) Suggestions for optimizing intervention content; (4) Expected outcomes and sustainability of the ATPISM strategy. The interview guide will be pre-tested with 3 experts (1 ophthalmologist, 1 school health administrator, 1 community health worker) to refine clarity and relevance, with revisions made to ambiguous questions. Between July and November 2025, two experienced qualitative researchers will be conducted semi-structured interviews via video conference. Each interview lasted approximately 30 min and was conducted by two experienced qualitative researchers: one researcher facilitated the interview based on the interview guide, while the other researcher recorded the conversation.

The Delphi technique is suitable for achieving consensus among experts within the same group, and it will be used in this study to help formulate intervention strategies [46]. A panel of experts specializing in the prevention of adolescent IS and myopia will be assembled, including specialists from education authorities, ophthalmology, rehabilitation medicine, school health and school physical education. All panel members possess extensive professional knowledge and practical experience. A detailed questionnaire will be designed to cover topics related to the prevention of IS and myopia, with an emphasis on preventive measures and screening methods. Three rounds of Delphi questionnaires will be completed using a five-point Likert scale for scoring. The expert opinions will be summarized and organized after each round. Following consolidation, the degree of consensus among the expert opinions will be evaluated again to identify issues requiring further discussion. In subsequent rounds, the results from the previous round will be provided to the experts to inform their responses to the next survey, enabling them to adjust their opinions and gradually reach a consensus. Once all the rounds have been completed, the project team will interpret and apply the expert opinions, identifying areas of agreement and disagreement, and formulate the ATPISM intervention measures through three rounds of feedback and consensus-building.

#### Quantitative data collection

All members participating in this study will undergo training and collect data using standardized assessment methods as specified in the study protocol. Assessors will be blinded to the allocation status of participants in the intervention and control groups. Participating schools will conduct data collection at three time points (baseline, mid-term, and terminal assessments) throughout the 12 months of the intervention period.

Demographic data will be collected once at baseline. Participants will be assigned a 5-digit identification code upon enrollment, which will link all study materials to the participant and replace their name. The study team leader will maintain a password protected list mapping these codes to participant names. Demographic information will be collected at baseline via an electronic questionnaire distributed through WeChat, gathering data on students’ names, birth dates, gender, ethnicity, grade level at enrollment, outdoor activity duration, and extracurricular training hours. Anthropometric measurements will include spinal and vision screenings, as well as body composition assessments. In this study, spinal and vision screenings will be conducted at baseline, mid-intervention, and 12 months post-intervention. Body composition measurements, behavioral indicators (reading/writing posture, outdoor activity, screen time, backpack habits, sleep duration, off-campus eye usage), and reports on depression and anxiety will be collected at baseline and 12 months post-intervention.

To evaluate potential moderating factors affecting the “ATPISM” intervention from multiple dimensions, questionnaires will be administered to schools, students, parents, and communities after 12 months of intervention based on the pilot study. The project team will provide detailed explanations of the questionnaires to the project leaders assigned to each school. These leaders will collaborate with homeroom teachers to guide and supervise the completion of questionnaires. Primary school students may complete the questionnaires with parental assistance.

The school questionnaire covers the following aspects: basic information, development of health education curriculum content, teacher training, physical environment setup and optimization, establishment of teacher-student health interaction mechanisms, health education, implementation of regular health screenings and health records, and construction of a comprehensive health network connecting schools, families, and communities. The student questionnaire focuses on health awareness cultivation, development of healthy behaviors, and self-monitoring of health. The parent questionnaire examines health awareness cultivation, home environment configuration, cooperation with health examinations, and participation in health education. The community questionnaire assesses community environment configuration and participation in health education.

### Outcomes

#### Primary outcome

The primary outcome will be the difference in IS and myopia screening positivity rates among children and adolescents between baseline and 12 months post-intervention. In spinal screening, individuals will be considered normal if there are no abnormalities in the general back examination, the Adam’s test is negative, and the trunk rotation angle (ATR) is < 5°. If any abnormalities will be present in the general back examination, the Adam’s test is positive, or the ATR is ≥ 5°, spinal motion tests will be repeated. An ATR of < 5° after retesting will indicate abnormal posture, while an ATR of ≥ 5°will indicate positive IS screening and will warrant follow-up at medical institutions. IS will be diagnosed when the Cobb angle measured on standing full-spine radiographs is ≥ 10° [47]. For visual acuity screening, distance vision will be assessed using computerized refraction without cycloplegia. Children and adolescents aged 6 years and older with uncorrected distance visual acuity of less than 5.0 and a spherical equivalent (SE) of no more than − 0.50 dioptres on cycloplegic refraction will be classified as myopia screening-positive [6].

#### Secondary outcomes

Body composition and behavioral indicators (e.g. reading/writing posture, outdoor activity, screen time, backpack habits, sleep duration and off-campus screen time) will be measured at baseline and 12 months post-intervention to assess differences in their effects. Self-reported depression and anxiety scale scores will also be measured at these time points. The specific measurement methods are as follows:

##### Body Composition Parameters

All participants must refrain from strenuous exercise the day prior to testing, and females should avoid testing during menstruation. To minimize the impact of digestive metabolism on measurement results, all patients will undergo body composition analysis using the body composition analyzer (InBody 260, South Korea) in the morning on an empty stomach while wearing light outer clothing. Measured body composition parameters include: body mass index (BMI), skeletal muscle mass (SMM), lean mass (LM), fat mass (FM), fat-free mass (FFM) and body fat percentage (%) [48].

##### Behavioral Indicators

The study will be conducted using self-reporting questionnaires completed by students and parents. The behavioral indicators to be collected in this research include reading/writing posture, outdoor activities, screen time, backpack habits, sleep duration and off-campus screen usage.

##### Depression and anxiety conditions

The Self-Rating Anxiety Scale (SAS) and Self-Rating Depression Scale (SDS) will be used to assess the severity of anxiety and depression symptoms, respectively, as well as changes in anxiety and depression states during treatment [49, 50].

### Statistical analyses

This study will be conducted using a mixed-methods design. For the qualitative analysis, two researchers will independently use NVivo 12.0 software to code and categories the content of expert interviews, having first verified the accuracy of the textual materials. They will then compare their coding results and discuss any discrepancies in order to reach a consensus. The statistical measures used in the Delphi study are measures of central tendency and dispersion [51]. Expert consensus on the formulation of the intervention strategy will be calculated using the median, the interquartile range (IQR) and the percentage of agreement. The median measures central tendency, while the IQR measures dispersion. For each expert opinion statement, the median, IQR and percentage of agreement will be calculated using SPSS 25.0 statistical software after each round. For the purposes of this study, “consensus” is defined as a median of 5 points, an IQR of ≤ 1 point, and ≥ 80% of scores being 4 or 5 points, while “no consensus” is defined as a median of 1 point, an IQR of ≤ 1 point, and ≥ 80% of scores being 1 or 2 points [52].

The quantitative component will utilize Excel 2021 software for organizing and cleaning invalid data, with SPSS 25.0 statistical software conducting data analysis. The baseline questionnaire survey data will employ descriptive analysis methods to describe the basic characteristics of children and adolescents in both groups. Count data will be indicated by frequency and percentage, with intergroup comparisons using the chi-square test; ordinal data will be compared using the rank sum test. Continuous normally distributed data will be expressed as (µx ± s), with between-group comparisons using paired t-tests and within-group pre-post comparisons using paired t-tests. Non-normally distributed data will be analyzed using the nonparametric Wilcoxon signed-rank test. To account for school clustering effects, mixed-effects models will be developed to compare group differences. Subgroup analyses will be conducted to identify interaction effects (stratification factor × group) and examine differences in intervention effects across strata. Additionally, Difference in Differences (DID) analysis will be employed to effectively evaluate the “ATPISM” intervention by comparing pre- and post-intervention changes between the intervention and control groups, resulting in the difference between the intervention group and the control group. To account for individual differences in teacher training effectiveness and parent supervision intensity, these factors will be included as moderating variables in mixed-effects models. Subgroup analyses will be conducted to compare intervention effects between groups with high/low implementation fidelity.

### Implementation evaluation

#### Study design

The RE-AIM framework, a public health model, will be used in Phase II of this study to evaluates the implementation with regard to acceptability, feasibility, adoption, usage, and maintenance of extending the ATPISM intervention to children and adolescents [38]. This process will commence in the preliminary phase of the intervention and will conclude 12 months after the intervention initiation. A variety of methods will be adopted for different participants, including individual and group interviews, assessment questionnaires, surveys of attendance and adoption rates, as well as intervention fidelity checklists or self-assessment forms.

#### Process evaluation

The RE-AIM dimensions are not arranged sequentially but are presented in the order they appear in the research.

##### Adoption

The adoption of the ATPISM intervention will be assessed among schools, students, parents and communities. The number of schools invited, those who declined, and those who ultimately enrolled to initiate the intervention will be recorded. The school adoption rate is defined as the percentage of schools with a trained health education team capable of implementing the intervention according to protocol, relative to the total number of schools.

##### Reach

We will evaluate the recruitment process to determine whether it meets the required target population size for this study. The number of participants screened, deemed eligible and successfully enrolled will be documented. It is defined as the percentage of enrolled participants relative to the number of eligible participants. Other evaluation metrics include participant characteristics and the intervention participation rate (i.e. the percentage of enrolled participants who complete the 12-month intervention).

##### Implementation

This phase will analyze the facilitating and barrier factors during the implementation of the ATPISM intervention. After the intervention commences, evaluators will observe it using the intervention fidelity checklist to assess the implementation of school health education for each teacher and their respective classes. The checklist will cover areas such as the development and application of the health education curriculum, student practice, teacher training, the physical school environment, campus health promotion activities, teacher-student health interactions and health service provision. Assessors will distribute self-assessment forms to parents and community members to rate the health environment and content of the education programme. For safety purposes, the research team will record all incidents (e.g. injuries or medical events directly related to the ATPISM intervention) through informal feedback from school staff, students, parents and community workers. Finally, students and parents will complete satisfaction surveys regarding the ATPISM intervention.

##### Effectiveness

Participant assessment data will be collected by assessors at baseline, at the mid-point, and 12 months after the intervention. The study will evaluate the effectiveness of the ATPISM programme in improving positive screening rates for IS, visual acuity, and body composition parameters, as well as behavioral indicators such as outdoor activity, screen time, fruit and vegetable intake, and consumption of high-quality protein. The study will also evaluate the programme effectiveness in reducing depression and anxiety, as measured by self-report scale scores, among children and adolescents. The incidence of adverse events will also be documented.

##### Maintenance

The maintenance of the ATPISM intervention will be reported from two perspectives (student and school) at the end of the study. This analysis will evaluate the intervention sustainability and its potential to become an integrated behavioral norm and practice within daily routines.

### Statistical analyses

Qualitative and quantitative analyses will be conducted using NVivo 12.0 and SPSS 25.0 statistical software. The significance level will be set at 0.05. Count data will be presented as frequencies and percentages. Continuous data following a normal distribution will be expressed as (µx ± s). Group comparisons will employ the chi-square (χ²) test, while ordinal data will be compared using the rank sum test. For the qualitative analysis, NVivo 12.0 software will be applied to code and categorize interview content regarding the perspectives of schools, students, parents, and community workers on the study components. The coded content will undergo thematic analysis.

### Pragmatic evaluation

The Pragmatic Explanatory Continuum Indicator Summary (PRECIS) model can help assess the pragmatic or explanatory nature of this study, ensuring the research design aligns more closely with its intended purpose. The updated PRECIS-2 online assessment tool (https://www.precis-2.org/) will be used to score nine dimensions: eligibility criteria, recruitment, setting, organization, flexibility of delivery, flexibility of adherence, follow-up, primary outcome, and primary analysis. Each dimension will be uniformly scored on a 1–5 scale within the PRECIS-2 wheel diagram. Lower scores indicate very explanatory, while higher scores denote very pragmatic [53].

## Discussion

This present study, guided by evidence-based research, will be designed to develop a comprehensive intervention strategy based on the WHO-advocated HPS framework. Centered on schools and supported by families and communities, it aims to create a school environment conducive to health and healthy behaviors to prevent the onset and progression of IS and myopia among children and adolescents. The implementation process, outcomes, and costs of this intervention strategy will be evaluated using the RE-AIM framework, with particular emphasis on assessing the impact on the sustainability of both the intervention process and the overall strategy.

Several factors, including schoolwork, daily lifestyle habits and physical activity, have been found to be closely associated with the development of IS and myopia [12, 14, 15, 17, 54]. Childhood and adolescence are periods of rapid physical development during which individuals are highly susceptible to environmental influences. Due to socioeconomic development and academic pressures, sedentary behaviors among children and adolescents have increased alongside reduced physical activity. Prolonged improper reading/writing postures and carrying heavy backpacks can cause fatigue and asymmetry in the strength of the paraspinal muscles, subsequently altering biomechanical structures and ultimately affecting spinal shape and function [55]. Myopia is closely related to the intensity of education. Prolonged reading and writing, along with increased screen time, have led to increase near-work duration [56]. A meta-analysis revealed that each additional 1 diopter-hour of weekly near work increases the incidence of myopia by 2% [57]. Previous studies consistently support the causal role of academic factors in myopia progression [16, 58]. The WHO recommends that children and adolescents engage in moderate-to-vigorous aerobic exercise for an average of 60 min daily to benefit their physical health [59]. Adequate physical activity, particularly outdoor activity, protects against poor skeletal and visual development. The spinal and visual health of children and adolescents is influenced by multiple factors. Implementing prevention and intervention strategies early in life to encourage positive attitudes and behaviors in this population can have a significant impact on their future health and well-being [15, 56]. This aligns with the HPS framework: healthier children achieve better educational outcomes, which in turn correlate more strongly with health in later life [27].

Therefore, this study aims to optimize and integrate key technologies developed earlier for the prevention of IS and myopia in children and adolescents. It will also develop a health education curriculum based on the core features of HPS for the prevention of IS and myopia, and provide training for teachers and community workers on identifying IS and its early symptoms, as well as guiding healthy behaviors. Attention will be paid to setting up and optimizing the physical environment of the school. This should include desk and chair height, lighting, space and equipment for physical activity, and green space. The values of health education should be promoted through publicity campaigns that emphasize building a school ethos and establishing teacher-student interactions. Regular spinal and vision screenings should be conducted for students, and one-on-one health profiles should be established. The school health education system should be developed to ensure health education is carried out properly. A health monitoring system should be set up to monitor students with IS and those at high risk. It should also provide health prescriptions for those in the healthy, high-risk and abnormal groups. Apart from schools, families and communities also have an important impact on the health of children and adolescents.

It is crucial to ensure the maintenance and replicability of intervention effectiveness. Therefore, this study will develop an evaluation model based on the RE-AIM framework. The model will assess the ATPISM intervention across five dimensions: reach, effectiveness, adoption, implementation and maintenance. Specifically, the evaluation will consider the intervention’s coverage and effectiveness, institutions’ willingness to adopt it, implementation outcomes, consistency with the project plan and maintenance of the ATPISM intervention after the study concludes [38]. The PRECIS model will then help to determine the practicality and interpretability of the study and enhance the intervention’s impact and sustainability. It will also provide evidence for exploring the long-term effectiveness of IS and myopia prevention interventions during childhood and adolescence and for improving health outcomes [60].

The present study has several strengths. Firstly, to our knowledge, this is the first time that the HPS framework has been applied in China to develop comprehensive intervention measures aimed at preventing two prevalent conditions that threaten the health of children and adolescents: IS and myopia. The school-centered approach emphasizes the establishment of foundational health education curricula and recognizes the importance of school-based health strategies, linkage with health services and collaborative relationships among schools, families and communities. Secondly, the study will use the RE-AIM framework to evaluate the feasibility and acceptability of the effectiveness of the ATPISM intervention. Additionally, the PRECIS model will be employed to clarify the study’s practicality and interpretability. While this research holds promise for advancing IS and myopia prevention among children and adolescents, several potential limitations should be noted. Firstly, the study population is not representative of the entire nation. Secondly, the findings may be limited and should be interpreted with caution.

There are several strengths of the present study. First, to our knowledge, this will be the first time in China that the HPS framework is applied to develop comprehensive intervention measures aimed at preventing IS and myopia. The school-centered approach not only emphasizes the establishment of foundational health education curricula but also recognizes the importance of school-based health strategies, the linkage with health services, and collaborative relationships among schools, families, and communities. Second, it will use the RE-AIM framework to assess the feasibility and acceptability of the ATPISM intervention’s effectiveness. Additionally, the PRECIS model will be employed to clarify the study’s practicality and interpretability. While this research holds promise for advancing IS and myopia prevention among children and adolescents, several potential limitations should be emphasized. The study population is not representative of the entire nation, and the findings may be limited and should be interpreted with caution.

In conclusion, this study is expected to address gaps in two areas. Firstly, the study aims to prevent IS and myopia simultaneously through the ATPISM intervention measures. Secondly, the study marks the first application of the HPS framework to reduce the incidence of IS and myopia. This approach provides a rational solution to the co-occurrence of IS and myopia among Chinese children and adolescents, while also advancing the establishment of HPS. The HPS model focuses on the health of children and adolescents, using schools as a platform to promote health, public health, education, social development and economic growth. Ultimately, the aim is to safeguard the health rights of children and adolescents and meet their health needs.

## Supplementary Information

Supplementary Material 1: SPIRIT 2013 Checklist: Recommended items to address in a clinical trial protocol and related documents*.

Supplementary Material 2: Ethical Approval Letter.

## Data Availability

The datasets generated and/or analyzed during the current study are not publicly available due confidentiality provisions in the consent process, but are available from the corresponding author on reasonable request.

## Abbreviations

IS: Idiopathic Scoliosis
HPS: Health Promoting School
WHO: World Health Organization
ATPISM: Appropriate Techniques for the Prevention of Idiopathic Scoliosis and Myopia
DID: Difference in Differences
RE-AIM: Reach, Effectiveness, Adoption, Implementation, Maintenance
SAS: The Self-Rating Anxiety Scale
SDS: Self-Rating Depression Scale
