# Supplementary material for: Effectiveness and implementation of an intervention strategy-based health promoting schools framework to reduce idiopathic scoliosis and myopia among children and adolescents in Shanghai, China: study protocol
Source: BMC Public Health. 2026 Jul 9;26:2865. doi: 10.1186/s12889-026-28184-5 (PMC13647753; doi:10.1186/s12889-026-28184-5)
Supplement: Supplementary file 1 [file 12889_2026_28184_MOESM2_ESM.pdf]

Ethical Review Approval Letter of Chongming Hospital Affiliated to Shanghai University of Medicine and Health Sciences

Disclaimer: This Ethics Committee is established and operates in accordance with relevant laws and regulations of the National Health Commission and NMPA. Its review and work processes are not influenced by any organization or individual outside the Ethics Committee.

No.: CMEC-2025-KT-51

**Project Title**	Effectiveness and implementation of an intervention strategy-based health promoting schools framework to reduce idiopathic scoliosis and myopia among children and adolescents in Shanghai, China: study protocol		
**Research Institution**	Chongming Hospital Affiliated to Shanghai University of Medicine and Health Sciences	**Principal Investigator**	Qing Du, Chief Physician
**Sponsor**	Not Applicable (N/A)		
**Review Date**	July 29, 2025	**Review Location**	/
**Review Type**	Initial review of scientific research project	**Review Method**	Expedited Review
**Investigational Drug/Device**	Not Applicable (N/A)		
**Clinical Study Phase**	□ Phase I □ Phase II □ Phase III □ Phase IV ■ Other: None		
**Review Documents**	1. Initial Review Application Form<br>2. List of Submitted Documents<br>3. PI Commitment and Conflict of Interest Disclosure<br>4. Research Project Summary<br>5. Study Protocol (V1.0, July 17, 2025)<br>6. Informed Consent Form (Subject, V1.0, July 17, 2025)<br>Informed Consent Form (Guardian, V1.0, July 17, 2025)<br>7. Curriculum Vitae of the Principal Investigator		
**Review Committee Members**	Juan Chang, Huixiang Wang		
**Review Opinion**	1. Following expedited review, the Ethics Committee confirms that the study is in compliance with relevant medical ethics laws and regulations; the review conclusion is: **Approved.**<br>2. Scheduled follow-up review of the study implementation:<br>□ No ■ Yes □ 3 months □ 6 months ■ 12 months<br><br>**Seal:** Approval Letter of Ethics Committee, Chongming Hospital Affiliated to Shanghai University of Medicine and Health Sciences.<br>**Date:** July 29, 2025		

**Note**	1. The study shall be conducted in strict accordance with the latest approved study protocol, and the health and rights of research subjects shall be protected in compliance with relevant clinical research regulations and guidelines.<br>2. Any content involving the export of human genetic resources or requiring special approval from relevant national departments shall be submitted to the competent authorities for approval and filed with this Ethics Committee prior to the study initiation.<br>3. This approval may be referenced by the Ethics Committees of other participating centers; prompt communication with this Ethics Committee is required if there are divergent opinions on the study review.<br>4. The Ethics Committee reserves the right to adjust the frequency of scheduled follow-up reviews based on the actual study progress.<br>5. This approval shall automatically become invalid if the study is not initiated within one year from the date of issuance.<br>6. Any modifications to the principal investigator, study protocol, informed consent forms, recruitment materials, etc., during the study must be submitted for amendment review and approved prior to implementation.<br>7. Serious adverse events and unexpected events affecting the risk-benefit ratio of the study must be reported to this Ethics Committee immediately.<br>8. The scheduled follow-up review report shall be submitted one month before the deadline in accordance with the review frequency specified by the Ethics Committee.<br>9. Any non-compliance or violations of ethical requirements must be reported to the Ethics Committee for review immediately.<br>10. This Ethics Committee shall be promptly notified if the clinical study is suspended or terminated early.<br>11. The final study report shall be submitted to this Ethics Committee for review upon the completion of the study.
**Ethics Committee Contact**	**Address:** No.309 Science and Education Building, No.25 Nanmen Road, Chongming District, Shanghai<br>**Postal Code:** 202150<br>**Telephone:** +86-21-69699328
