# Supplementary material for: Effectiveness and implementation of an intervention strategy-based health promoting schools framework to reduce idiopathic scoliosis and myopia among children and adolescents in Shanghai, China: study protocol
Source: BMC Public Health. 2026 Jul 9;26:2865. doi: 10.1186/s12889-026-28184-5 (PMC13647753; doi:10.1186/s12889-026-28184-5)
Supplement: Supplementary file 2 [file 12889_2026_28184_MOESM1_ESM.doc]

SPIRIT 2013 Checklist: Recommended items to address in a clinical trial protocol and related documents*

| Section/item | ItemNo | Description |
| --- | --- | --- |
| **Administrative information** | | |
| Title | 1 | Descriptive title identifying the study design, population, interventions, and, if applicable, trial acronym  ***Page 1.*** |
| Trial registration | 2a | Trial identifier and registry name. If not yet registered, name of intended registry  ***Page 2, Trial registration.*** |
|  | 2b | All items from the World Health Organization Trial Registration Data Set  ***Not applicable.*** |
| Protocol version | 3 | Date and version identifier  ***Not applicable.*** |
| Funding | 4 | Sources and types of financial, material, and other support  ***Page 35, Funding.*** |
| Roles and responsibilities | 5a | Names, affiliations, and roles of protocol contributors  ***Page 35, Authors’ Contributions.*** |
|  | 5b | *Name and contact information for the trial sponsor*  ***Page 35, Funding.*** |
|  | 5c | Role of study sponsor and funders, if any, in study design; collection, management, analysis, and interpretation of data; writing of the report; and the decision to submit the report for publication, including whether they will have ultimate authority over any of these activities  ***Page 35, Authors’ Contributions.*** |
|  | 5d | Composition, roles, and responsibilities of the coordinating centre, steering committee, endpoint adjudication committee, data management team, and other individuals or groups overseeing the trial, if applicable (see Item 21a for data monitoring committee)  ***Not applicable.*** |
| Introduction |  |  |
| Background and rationale | 6a | Description of research question and justification for undertaking the trial, including summary of relevant studies (published and unpublished) examining benefits and harms for each intervention  ***Page 2-7, Introduction.*** |
|  | 6b | Explanation for choice of comparators  ***Page 7, Effectiveness evaluation.*** |
| Objectives | 7 | Specific objectives or hypotheses  ***Page 6-7, Introduction.*** |
| Trial design | 8 | *Description of trial design including type of trial (eg, parallel group, crossover, factorial, single group), allocation ratio, and framework (eg, superiority, equivalence, noninferiority, exploratory)*  ***Page 7, Study setting.*** |
| Methods: Participants, interventions, and outcomes | | |
| Study setting | 9 | Description of study settings (eg, community clinic, academic hospital) and list of countries where data will be collected. Reference to where list of study sites can be obtained  ***Page 7, Effectiveness evaluation.*** |
| Eligibility criteria | 10 | Inclusion and exclusion criteria for participants. If applicable, eligibility criteria for study centres and individuals who will perform the interventions (eg, surgeons, psychotherapists)  ***Page 9, Eligibility criteria.*** |
| Interventions | 11a | Interventions for each group with sufficient detail to allow replication, including how and when they will be administered  ***Page 11, Intervention.*** |
|  | 11b | Criteria for discontinuing or modifying allocated interventions for a given trial participant (eg, drug dose change in response to harms, participant request, or improving/worsening disease)  ***N/A.*** |
|  | 11c | Strategies to improve adherence to intervention protocols, and any procedures for monitoring adherence (eg, drug tablet return, laboratory tests)  ***Page 15, Quality control.*** |
|  | 11d | Relevant concomitant care and interventions that are permitted or prohibited during the trial  ***Page 11, Intervention.*** |
| Outcomes | 12 | Primary, secondary, and other outcomes, including the specific measurement variable (eg, systolic blood pressure), analysis metric (eg, change from baseline, final value, time to event), method of aggregation (eg, median, proportion), and time point for each outcome. Explanation of the clinical relevance of chosen efficacy and harm outcomes is strongly recommended  ***Page 21, Outcomes.*** |
| Participant timeline | 13 | Time schedule of enrolment, interventions (including any run-ins and washouts), assessments, and visits for participants. A schematic diagram is highly recommended (see Figure)  ***Page 7, Effectiveness evaluation, and Figure 2.*** |
| Sample size | 14 | Estimated number of participants needed to achieve study objectives and how it was determined, including clinical and statistical assumptions supporting any sample size calculations  ***Page 8, Sample size.*** |
| Recruitment | 15 | Strategies for achieving adequate participant enrolment to reach target sample size  ***Page 9, Eligibility criteria.*** |
| **Methods: Assignment of interventions (for controlled trials)** | | |
| Allocation: |  |  |
| Sequence generation | 16a | Method of generating the allocation sequence (eg, computer-generated random numbers), and list of any factors for stratification. To reduce predictability of a random sequence, details of any planned restriction (eg, blocking) should be provided in a separate document that is unavailable to those who enrol participants or assign interventions  ***Page 6, Randomization and Blinding.*** |
| Allocation concealment mechanism | 16b | Mechanism of implementing the allocation sequence (eg, central telephone; sequentially numbered, opaque, sealed envelopes), describing any steps to conceal the sequence until interventions are assigned  ***Not applicable.*** |
| Implementation | 16c | Who will generate the allocation sequence, who will enrol participants, and who will assign participants to interventions  ***Page 8, Randomization and Blinding.*** |
| Blinding (masking) | 17a | Who will be blinded after assignment to interventions (eg, trial participants, care providers, outcome assessors, data analysts), and how  ***Page 8, Randomization and Blinding.*** |
|  | 17b | If blinded, circumstances under which unblinding is permissible, and procedure for revealing a participant’s allocated intervention during the trial  ***Page 8, Randomization and Blinding.*** |
| **Methods: Data collection, management, and analysis** | | |
| Data collection methods | 18a | Plans for assessment and collection of outcome, baseline, and other trial data, including any related processes to promote data quality (eg, duplicate measurements, training of assessors) and a description of study instruments (eg, questionnaires, laboratory tests) along with their reliability and validity, if known. Reference to where data collection forms can be found, if not in the protocol  ***Page 17, Data Collection.*** |
|  | 18b | Plans to promote participant retention and complete follow-up, including list of any outcome data to be collected for participants who discontinue or deviate from intervention protocols  ***Page 17, Data Collection.*** |
| Data management | 19 | Plans for data entry, coding, security, and storage, including any related processes to promote data quality (eg, double data entry; range checks for data values). Reference to where details of data management procedures can be found, if not in the protocol  ***Page 17, Data Collection.*** |
| Statistical methods | 20a | Statistical methods for analysing primary and secondary outcomes. Reference to where other details of the statistical analysis plan can be found, if not in the protocol  ***Page 23, Statistical analysis.*** |
|  | 20b | Methods for any additional analyses (eg, subgroup and adjusted analyses)  ***Page 23, Statistical analysis.*** |
|  | 20c | Definition of analysis population relating to protocol non-adherence (eg, as randomised analysis), and any statistical methods to handle missing data (eg, multiple imputation)  ***Page 23, Statistical analysis.*** |
| **Methods: Monitoring** | | |
| Data monitoring | 21a | Composition of data monitoring committee (DMC); summary of its role and reporting structure; statement of whether it is independent from the sponsor and competing interests; and reference to where further details about its charter can be found, if not in the protocol. Alternatively, an explanation of why a DMC is not needed  ***Page 34, Competing interests.*** |
|  | 21b | Description of any interim analyses and stopping guidelines, including who will have access to these interim results and make the final decision to terminate the trial  ***Not applicable.*** |
| Harms | 22 | Plans for collecting, assessing, reporting, and managing solicited and spontaneously reported adverse events and other unintended effects of trial interventions or trial conduct  ***Page 9, Eligibility criteria.*** |
| Auditing | 23 | *Frequency and procedures for auditing trial conduct, if any, and whether the process will be independent from investigators and the sponsor*  ***Not applicable.*** |
| Ethics and dissemination | | |
| Research ethics approval | 24 | Plans for seeking research ethics committee/institutional review board (REC/IRB) approval  ***Page 34, Ethical approval and consent to participate.*** |
| Protocol amendments | 25 | *Plans for communicating important protocol modifications (eg, changes to eligibility criteria, outcomes, analyses) to relevant parties (eg, investigators, REC/IRBs, trial participants, trial registries, journals, regulators)*  ***Not applicable.*** |
| Consent or assent | 26a | Who will obtain informed consent or assent from potential trial participants or authorised surrogates, and how (see Item 32)  ***Page 9, Eligibility criteria.*** |
|  | 26b | Additional consent provisions for collection and use of participant data and biological specimens in ancillary studies, if applicable  ***Page 9, Eligibility criteria.*** |
| Confidentiality | 27 | How personal information about potential and enrolled participants will be collected, shared, and maintained in order to protect confidentiality before, during, and after the trial  ***Page 17, Data Collection.*** |
| Declaration of interests | 28 | *F*inancial and other competing interests for principal investigators for the overall trial and each study site  ***Page 34,*** ***Competing interests.*** |
| Access to data | 29 | Statement of who will have access to the final trial dataset, and disclosure of contractual agreements that limit such access for investigators  ***Page 34, Availability of data and materials.*** |
| Ancillary and post-trial care | 30 | Provisions, if any, for ancillary and post-trial care, and for compensation to those who suffer harm from trial participation  ***Not applicable.*** |
| Dissemination policy | 31a | Plans for investigators and sponsor to communicate trial results to participants, healthcare professionals, the public, and other relevant groups (eg, via publication, reporting in results databases, or other data sharing arrangements), including any publication restrictions  ***Not applicable.*** |
|  | 31b | Authorship eligibility guidelines and any intended use of professional writers  ***Not applicable.*** |
|  | 31c | Plans, if any, for granting public access to the full protocol, participant-level dataset, and statistical code  ***Not applicable.*** |
| Appendices |  |  |
| Informed consent materials | 32 | Model consent form and other related documentation given to participants and authorised surrogates  ***Not applicable.*** |
| Biological specimens | 33 | Plans for collection, laboratory evaluation, and storage of biological specimens for genetic or molecular analysis in the current trial and for future use in ancillary studies, if applicable  ***Not applicable.*** |

*It is strongly recommended that this checklist be read in conjunction with the SPIRIT 2013 Explanation & Elaboration for important clarification on the items. Amendments to the protocol should be tracked and dated. The SPIRIT checklist is copyrighted by the SPIRIT Group under the Creative Commons “[Attribution-NonCommercial-NoDerivs 3.0 Unported](http://www.creativecommons.org/licenses/by-nc-nd/3.0/)” license.
